# Impact of 1 mm difference in interelectrode spacing on local electrograms and voltage/activation maps

**DOI:** 10.1093/europace/euaf135

**Published:** 2025-07-24

**Authors:** Junji Yamaguchi, Masateru Takigawa, Masahiko Goya, Hidehiro Iwakawa, Iwanari Kawamura, Ryosuke Kato, Masaki Honda, Miho Negishi, Ryo Tateishi, Tasuku Yamamoto, Takashi Ikenouchi, Kentaro Goto, Takatoshi Shigeta, Takuro Nishimura, Tomomasa Takamiya, Susumu Tao, Sayaka Suzuki, Takehiro Iwanaga, Shinsuke Miyazaki, Tetsuo Sasano

**Affiliations:** Department of Cardiovascular Medicine, Institute of Science Tokyo, 1-5-45 Yushima, Bunkyo-ku, Tokyo 113-8510, Japan; Department of Clinical and Diagnostic Laboratory Science, Institute of Science Tokyo, Tokyo, Japan; Department of Cardiovascular Medicine, Institute of Science Tokyo, 1-5-45 Yushima, Bunkyo-ku, Tokyo 113-8510, Japan; Division of Advanced Arrhythmia Research, Institute of Science Tokyo, Tokyo, Japan; Department of Cardiovascular Medicine, Institute of Science Tokyo, 1-5-45 Yushima, Bunkyo-ku, Tokyo 113-8510, Japan; Department of Cardiovascular Medicine, Akita University Graduate School of Medicine, Akita, Japan; Department of Cardiovascular Medicine, Institute of Science Tokyo, 1-5-45 Yushima, Bunkyo-ku, Tokyo 113-8510, Japan; Department of Cardiovascular Medicine, Akita University Graduate School of Medicine, Akita, Japan; Department of Cardiovascular Medicine, Institute of Science Tokyo, 1-5-45 Yushima, Bunkyo-ku, Tokyo 113-8510, Japan; Department of Cardiovascular Medicine, Institute of Science Tokyo, 1-5-45 Yushima, Bunkyo-ku, Tokyo 113-8510, Japan; Department of Cardiovascular Medicine, Institute of Science Tokyo, 1-5-45 Yushima, Bunkyo-ku, Tokyo 113-8510, Japan; Department of Cardiovascular Medicine, Institute of Science Tokyo, 1-5-45 Yushima, Bunkyo-ku, Tokyo 113-8510, Japan; Department of Cardiovascular Medicine, Institute of Science Tokyo, 1-5-45 Yushima, Bunkyo-ku, Tokyo 113-8510, Japan; Department of Cardiovascular Medicine, Institute of Science Tokyo, 1-5-45 Yushima, Bunkyo-ku, Tokyo 113-8510, Japan; Department of Cardiovascular Medicine, Institute of Science Tokyo, 1-5-45 Yushima, Bunkyo-ku, Tokyo 113-8510, Japan; Department of Cardiovascular Medicine, Institute of Science Tokyo, 1-5-45 Yushima, Bunkyo-ku, Tokyo 113-8510, Japan; Department of Cardiovascular Medicine, Institute of Science Tokyo, 1-5-45 Yushima, Bunkyo-ku, Tokyo 113-8510, Japan; Department of Cardiovascular Medicine, Institute of Science Tokyo, 1-5-45 Yushima, Bunkyo-ku, Tokyo 113-8510, Japan; Japan Small Animal Medical Center, Saitama, Japan; Center for Experimental Animals, Institute of Science Tokyo, Tokyo, Japan; Department of Cardiovascular Medicine, Institute of Science Tokyo, 1-5-45 Yushima, Bunkyo-ku, Tokyo 113-8510, Japan; Division of Advanced Arrhythmia Research, Institute of Science Tokyo, Tokyo, Japan; Department of Cardiovascular Medicine, Institute of Science Tokyo, 1-5-45 Yushima, Bunkyo-ku, Tokyo 113-8510, Japan

**Keywords:** Catheter ablation, OCTARAY, Electrogram mapping, Low-voltage zones, Gap detection

Multipolar mapping catheters can provide higher-resolution maps compared to ablation catheters,^1,2^ making them useful for gap detection and substrate characterization, which significantly influence treatment outcomes.^3–5^ This advantage is due to the smaller electrodes and closer interelectrode spacing, which increase the likelihood of recording near-field electrograms (EGMs). The relationship between interelectrode spacing and EGMs has been explored in previous studies,^6–9^ but little is known about the effects of interelectrode spacing of submillimetre electrode on local EGMs and voltage/activation maps. In this *in vivo* swine study, we compared two OCTARAY™ catheter configurations that differed only in centre-to-centre spacing (2 vs. 3 mm), while keeping the electrode size constant (0.5 mm), to determine how a 1 mm spacing difference affects unipolar and bipolar EGMs, low-voltage zone (LVZ) delineation, and activation-map gap detection.

Seven male swine (2.8–4.0 months; 48–56 kg) were anaesthetized with intramuscular ketamine (20 mg/kg), acepromazine (0.1 mg/kg), and buprenorphine (20 µg/kg), then intubated under intravenous propofol (2 mg/kg) and ventilated with 2–3% isoflurane. Femoral venous and arterial sheaths provided access for mapping and ablation catheters, as well as fluid and drug administration. A linear, point-by-point radiofrequency ablation was delivered along the posterior right atrial wall, intentionally leaving median 2^1,2^ gaps to evaluate the catheters’ ability to detect conduction gaps. Endocardial maps were created during coronary sinus ostial pacing on a CARTO 3 system using both OCTARAY configurations. Bipolar signals were band-pass filtered at 16–500 Hz and unipolar at 2–240 Hz. Bipolar LVZs were defined as contiguous regions with peak-to-peak amplitude ≤ 0.5 mV, and unipolar LVZs ≤ 2.5 mV. Two experienced electrophysiologists (J.Y., M.T.) blinded to catheter spacing independently measured LVZ areas and gap detection.

To directly evaluate the impact of interelectrode spacing on raw EGMs, epicardial mapping was conducted in three swine via median sternotomy, with potentials recorded at 30–35 distinct epicardial sites per animal (*Figure 1A*). As simultaneous deployment of both OCTARAY™ catheters (2 and 3 mm spacings) was technically unfeasible, EGMs were sequentially recorded at identical sites using each catheter. During each recording, a DECANAV™ catheter was positioned immediately adjacent to the OCTARAY™ to acquire reference EGMs. The relative differences in EGM parameters between the 2 and 3 mm spacing configurations were indirectly derived using the DECANAV™ signals as a normalization reference. Signals were sampled on LabSystem Pro with unipolar filters set at 0.1–100 Hz and bipolar at 30–500 Hz as previously described.^10^ We quantified unipolar voltage and duration at distal and proximal electrodes, bipolar voltage, near-field potential duration (the sharp high-frequency component), and far-field duration (the full potential width). Data are reported as mean ± SD, and comparisons used unpaired two-tailed *t*-tests (significance *P* < 0.05).

**Figure 1:**
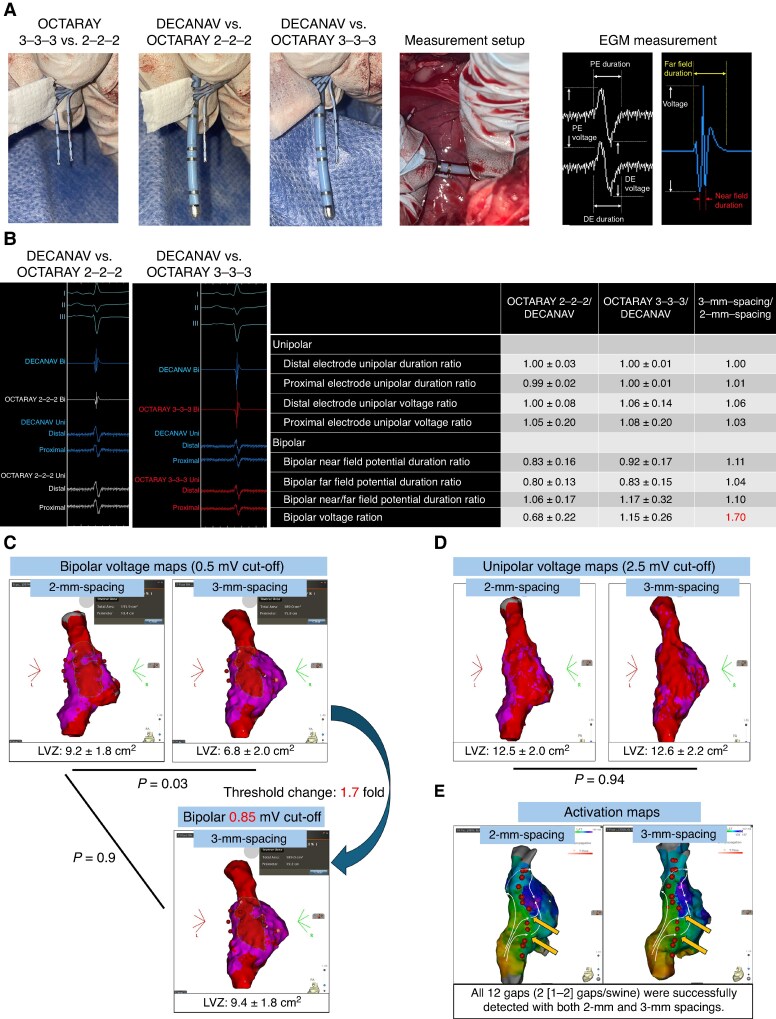
(*A*) Photographs of the OCTARAY 3-3-3, OCTARAY 2-2-2, and DECANAV catheters, together with schematic definitions of bipolar and unipolar electrogram (EGM) measurements. PE, proximal electrode; DE, distal electrode. (*B*) Left: representative unipolar and bipolar EGMs recorded with OCTARAY 2-2-2 and 3-3-3. Right: summary table of EGM measurements for OCTARAY 2-2-2 vs. DECANAV and OCTARAY 3-3-3 vs. DECANAV, plus the calculated OCTARAY 3-3-3/OCTARAY 2-2-2 amplitude ratio. (*C*) Bipolar voltage mapping. Using the conventional 0.5 mV cut-off, the low-voltage zone (LVZ) was significantly smaller with 3 mm interelectrode spacing than with 2 mm (*P* = 0.03). When the 3 mm spacing cut-off was adjusted to 0.85 mV, LVZ areas became nearly identical between configurations (*P* = 0.90). (*D*) Unipolar voltage mapping. At a cut-off of 2.5 mV, LVZ areas did not differ between 2 and 3 mm spacing catheters (*P* = 0.94). (*E*) Activation map during pacing from the low-lateral right atrium (RA). Three converging wavefronts were observed: two traversing conduction gaps (arrows pointing to the right atrium) and one propagating anteriorly. All gaps were detected equally well with both 2 and 3 mm spacing configurations, and no qualitative differences in activation pattern were noted.

Across 100 epicardial sites, unipolar EGMs were identical between the 2 mm (OCTARAY 2-2-2) and 3 mm (OCTARAY 3-3-3) spacings (*Figure 1B*). Distal voltages were 8.3 ± 1.1 vs. 8.3 ± 1.2 mV (normalized to DECANAV: 1.00 ± 0.08 vs. 1.06 ± 0.14; *P* = 0.78), and proximal voltages 8.6 ± 1.0 vs. 8.7 ± 0.9 mV (normalized: 1.05 ± 0.20 vs. 1.08 ± 0.20; *P* = 0.91). Distal unipolar durations were 68.9 ± 5.4 vs. 67.6 ± 5.2 ms (normalized: 1.00 ± 0.03 vs. 1.00 ± 0.01; *P* = 0.94), and proximal durations 68.9 ± 5.4 vs. 67.6 ± 5.2 ms (normalized: 0.99 ± 0.02 vs. 1.00 ± 0.01; *P* = 0.86). Bipolar near-field duration measured 7.8 ± 1.0 vs. 6.9 ± 0.9 ms (normalized: 0.83 ± 0.16 vs. 0.92 ± 0.17; *P* < 0.01), while far-field duration was 49.0 ± 5.0 vs. 46.2 ± 4.8 ms (normalized: 0.80 ± 0.13 vs. 0.83 ± 0.15; *P* < 0.05). The near-to-far field duration ratio increased modestly at 3 mm (0.15 ± 0.04 vs. 0.13 ± 0.03; normalized: 1.06 ± 0.17 vs. 1.17 ± 0.32; *P* < 0.001). Most strikingly, bipolar voltage rose from 2.6 ± 1.6 to 3.4 ± 1.5 mV, with normalized values increasing 1.7-fold (0.68 ± 0.22 vs. 1.15 ± 0.26; *P* < 0.001). These results confirm that unipolar signals are spacing-independent, whereas wider electrode spacing modestly prolongs bipolar durations and substantially amplifies bipolar voltage.

Endocardial voltage mapping using a 0.5 mV bipolar cut-off revealed significantly smaller LVZs with 3 vs. 2 mm spacing (6.8 ± 1.2 vs. 9.2 ± 1.5 cm²; *P* = 0.03) (*Figure 1C*). Adjusting the 3 mm cut-off to 0.85 mV (×1.7) equalized LVZ areas (9.4 ± 1.4 vs. 9.2 ± 1.5 cm²; *P* = 0.90). Unipolar LVZs (2.5 mV cut-off) were comparable (12.6 ± 2.0 vs. 12.5 ± 2.1 cm²; *P* = 0.94) (*Figure 1D*). Activation mapping identified all seven intentional gaps with both spacings (12/12; 100%; *P* = 1.00) (*Figure 1E*).

A 1 mm increase in interelectrode spacing (with 0.5 mm electrodes) resulted in a 1.7-fold rise in bipolar voltage, while unipolar EGMs, spacing-adjusted bipolar LVZ assessment, and activation gap detection remained consistent. The 3 mm spacing likely captures more far-field signals, increasing bipolar amplitude and slightly prolonging durations. Clinically, applying uniform bipolar cut-offs across catheters with differing spacings may underestimate arrhythmogenic substrate with wider-spacing designs. Scaling voltage thresholds proportionally to spacing (e.g. 0.85 mV for 3 mm) enables consistent LVZ delineation across catheters. As unipolar mapping and gap detection remain unaffected by minor spacing differences, they offer reliable guidance regardless of catheter geometry.

A key limitation is the small epicardial sample size due to animal tolerability. However, EGMs were collected from multiple sites and time points, showing consistent trends across all swine, which we consider sufficient for analysis.

In summary, even a small 1 mm change in interelectrode spacing significantly alters bipolar voltage and LVZ measurements without impairing unipolar signals or gap detection. Adopting catheter-specific, spacing-adjusted bipolar cut-offs is essential for accurate substrate mapping and optimized ablation guidance.

## Data Availability

The data underlying this article will be shared on reasonable request to the corresponding author.
